# Evaluation of the masticatory muscle function, physiological sleep variables, and salivary parameters after electromechanical therapeutic approaches in adult patients with Down syndrome: a randomized controlled clinical trial

**DOI:** 10.1186/s13063-019-3300-0

**Published:** 2019-04-11

**Authors:** Lilian Chrystiane Giannasi, Marignês T. S. Dutra, Vera L. S. Tenguan, Gabriela P. Mancilha, Gabriela R. C. Silva, Elaine Fillietaz-Bacigalupo, Daniel B. da Silva, Fabiano Politti, Sergio R. Nacif, Ezequiel F. de Oliveira, João C. da Rocha, Carolina T. Rocha, Mateus M. Romero, Claudia S. de Oliveira, Luis V. F. de Oliveira, Sigmar de Mello Rode, Cristiane Yumi Koga-Ito, Jose B. O. Amorim, Miguel A. C. Salgado, Mônica F. Gomes

**Affiliations:** 1 Center of Biosciences Applied to Patients with Special Health Care Needs (CEBAPE), Institute of Science and Technology, São José dos Campos Campus, São Paulo State University—UNESP, R: Esperança 265, São Paulo, SP Brazil; 2 Metropolitan University of Santos, Santos, Brazil; 3 University Center of Anápolis—UniEvangélica, Anápolis, Brazil; 4 Nove de Julho University, São Paulo, Brazil; 5 Hospital do Servidor Público Estadual de São Paulo (IAMSPE-HSPE), São Paulo, Brazil; 6 Discipline of Pediatric Dentistry Institute of Science and Technology, São Paulo, Brazil

**Keywords:** Down syndrome, Sleep apnea, Polysomnography, Masticatory muscles, Electromyography, Oral appliance, Saliva, Cortisol

## Abstract

**Background:**

There are many comorbidities associated with Down syndrome (DS), including obstructive sleep apnea (OSA) and masticatory muscle alteration. Muscular hypotonia, in particular, of the masticatory and oropharyngeal muscles is one of the main characteristics of individuals with DS, resulting in impairments of speech, swallowing, and mastication in these individuals. In addition, total or partial obstruction of the airways during sleep can occur due to pharyngeal hypotonia, leading to snoring and to OSA. This progressive respiratory disorder is associated with a high risk of morbidity and mortality in individuals with DS. The aim of this research is to assess the therapeutic effects of surface neuromuscular electrical stimulation (NMES), the mastication apparatus (MA), and a mandibular advancement oral appliance (OA_m_) with an embedded thermosensitive microchip on the functions of masticatory muscles (bilateral masseter and temporal muscles), physiological sleep variables, and salivary parameters in adult patients with DS.

**Methods:**

The patients with DS will be randomly selected and divided into three groups (DS-NMES, DS-MA, and DS-OA_m_) with a minimum of 10 patients in each group. A thermosensitive microchip will be embedded in the OA_m_ to record its compliance. The therapeutic effects on masticatory muscle function will be investigated through electromyography, a caliper, and a force-transducer device; the sleep variables, in turn, will be evaluated by means of polysomnography. The physicochemical and microbiological properties of the saliva will also be analyzed, including the salivary flow, viscosity, buffer capacity, cortisol levels (susceptibility to psychological and/or physical stress), and *Pseudomonas aeruginosa* levels (risk of aspiration pneumonia) in these patients. The methods determined for this study will be carried out prior to and after 2 months of the recommended therapies.

**Discussion:**

The primary outcomes would be the improvement and/or reestablishment of the function of masticatory muscles and the physiological sleep variables in this target public since individuals with DS commonly present generalized muscular hypotonia and dysfunction of the oropharyngeal musculature. As a secondary outcome indicator, the impact of the applied therapies (NMES, MA, and OA_m_) on the salivary microbiological and physicochemical properties in DS individuals will also be assessed. Furthermore, the compliance of OA_m_ usage will be measured through a thermosensitive microchip.

**Trial registration:**

Registro Brasileiro de Ensaios Clínicos, RBR-3qp5np*.* Registered on 20 February 2018.

**Electronic supplementary material:**

The online version of this article (10.1186/s13063-019-3300-0) contains supplementary material, which is available to authorized users.

## Background

The chromosomal alteration caused by trisomy 21, known as Down syndrome (DS), is the main cause of mental impairment and is also related to several psychosocial limitations and comorbidities. Its prevalence in the general population is 1 per 600–800 live births. Nevertheless, if these conditions are well managed, these individuals may reach their full potential regarding mental and physical health, which allows their inclusion in society [1].

This condition is usually accompanied by mental retardation at many levels, cardiovascular disease, thyroid dysfunction, upper airway obstruction, respiratory breathing disorders, reduced size of maxillary and mandibular bones, muscular hypotonia, breathing through the mouth, risk for aspiration pneumonia, and low immunity, among other features [2–4].

Considering that muscle hypotonia is one of the main characteristics in individuals with DS that can affect any muscle in the body, the loss or reduction of upper airway (UA) muscle tone, including the oropharynx, velopharynx, hypopharynx, and tongue base muscles, may lead to partial/total obstruction causing obstructive sleep apnea (OSA) [5, 6].

In addition, the diminished activity of the genioglossus and other dilator muscles combined with an altered UA anatomy are probably the main cause of obstructive events [7–10]. This disease is a growing public health issue and is associated with a high risk of morbidity and mortality [4].

The genioglossus, a representative dilator muscle of the upper airways, plays an important role in the pathophysiology of OSA. In apneic individuals, a reduction in the activity of several muscles of the UA is observed. To compensate for this, some dilator muscles, along with the genioglossus, may increase activity in response to an increased negative pressure. When the dilator muscles reflex is not sufficient to compensate the negative pressure, the upper airway may collapse. This fact was demonstrated by Jordan et al. [7], who observed that when apneic individuals spontaneously overcame their tendency for UA collapse and had stable breathing during sleep, the genioglossus muscle was more active than during respiratory events, demonstrating that augmentation of genioglossus muscle tone is associated with spontaneous periods of stable flow-limited breathing in apneic individuals.

An animal model study and an investigation with healthy individuals have shown that greater respiratory resistance induces increased muscle activity of the orofacial muscles, bringing the tongue forward and opening the mouth for better respiration [9, 10], but in OSA individuals the amount of muscle reflex may be not enough to prevent the disease. The exact mechanism of OSA is not totally clarified and finding new pathways to sufficiently increase the activity of upper airway muscle is necessary, including in persons with disability.

The prevalence of obstructive sleep apnea (OSA) in adults with DS is still unknown, but in children the prevalence is around 66% [5]. Adults with DS will commonly present signs of OSA, hypoxemia, and sleep fragmentation [6]. Symptoms of OSA include snoring, excessive daytime somnolence, fatigue, nonrestorative sleep, memory lapses, cognitive impairment, and mood alteration. If OSA remains nontreated, various comorbidities may take place, such as hypertension, stroke, insulin resistance, metabolic alteration, and depression/anxiety.

Studies have demonstrated satisfactory results through oropharyngeal exercises in adult patients with no disability. However, a portion of people with disability has compromised cognition, which would prevent this population performing the oropharyngeal therapy correctly [11, 12]. Thus, new therapies are necessary in order to allow an improvement in upper airway muscles in this population.

There are few research studies correlating the masticatory muscle functions and sleep disorders in adult patients with DS. Thus, previous studies have suggested that new therapeutic approaches are promising options to improve masticatory muscle function and to treat sleep disorders, including sleep bruxism and OSA. These investigations have demonstrated satisfactory results in patients with disabilities who presented concomitant unbalanced masseter and temporalis muscle patterns, sleep bruxism, and OSA [13–15].

One of these studies used surface electromyography (sEMG) to evaluate pre and post treatment of masticatory muscle functions of severe sleep bruxism with mechanical stimulus in a child with cerebral palsy. The results showed a reduction of sleep bruxism and electrical activity balance in the masticatory muscles after mechanical stimulus with the mastication instrument Hiperboloide® [13]. It is important to provide the brief information that this mastication instrument was also named hyperbola, hiperbolide, or hyperboloid in different previous studies, but all are the same device [13, 16]. In other work, Giannasi et al. [14] demonstrated that it is possible to achieve electrical activity balance in the masticatory muscles and a reduction in OSA severity using neuromuscular electrical stimulation (NMES) therapy through sEMG and polysomnography (PSG). The authors showed a statistically significant reduction in snoring and respiratory events after the treatment with NMES in adult cerebral palsy. Also, parents have reported a visible reduction of chewing and drooling in daily routine function [8]. The sustained hypothesis for this positive result is that the neuroelectrical stimulus has reverberated on all muscular chains from activation of the masseter and temporalis muscles, and indirectly improved the oropharyngeal, velopharyngeal, and tongue base tonus, which also resulted in a decrease of respiratory events.

NMES has been used for maintenance of muscle mass, strength during long periods of immobilization, selective muscle retraining, and muscle strength. It is not only used to treat spasticity [17]. So, it may also be used to treat hypotonia, as seen in a recent study by Pinheiro et al. [18] that showed positive results in the treatment of weakness muscle with NMES in DS patients. The authors found significant differences in the muscle behavior during breathing, swallowing, and mastication function, pre and post treatment with NMES, applied on the masseter [10]. Based on these outcomes, they inferred that muscle fibers can modify their physiologic and biochemical characteristics according to the stimulus to which they are submitted. Such adaptive capacity involving several components of the fiber corresponds to muscular plasticity. Fraga et al. [19] stated that the mechanism of muscular contraction may occur due to a controlled and coordinated voluntary command by the brain, or due to an involuntary contraction induced by an external electrical stimulus. Abdel-Moty et al. [20] and Tong et al. [21] also showed positive results with the use of NMES to treat hypotonia and to promote change in the muscle fiber composition. Thus, the therapeutic options (i.e. mechanical and electrical stimulation) and the relationship between the masticatory muscles and the respiratory function are still not well studied and need to be better understood and clarified.

Another usual option to treat snoring, OSA, and associated symptoms is the mandibular advancement oral appliance (OA_m_). The OA_m_ is a therapy to manage OSA and refers to the mandibular advancement. Thus, the OA_m_ function is to protrude and to stabilize the mandible in order to maintain the patency of the upper airway (UA) during sleep. The action mechanism of the OA_m_ aims to increase the volume and to reduce the collapsibility of the UA with the mandibular advancement. The OA_m_ will protrude the mandible and the tongue, since the genioglossus that is one of the tongue’s major muscles is attached to the lingual surface of the anterior mandibular arch. The increase in the upper airway dimension occurs especially in the velopharyngeal area, which is greater than in the oropharyngeal and hypopharyngeal sites. This is likely a consequence of stretching the soft tissue connecting the soft palate, lateral pharynx, tongue, and palatopharyngeal arches. All of this upper airway alteration will promote the reduction and/or elimination of snoring and other respiratory events, as well as associated symptoms. In patients with no disability, the OA_m_ is a safe and effective treatment for OSA, with positive results [22]. Also, patients report that after OA_m_ usage they observed an improvement in word spelling in the daily clinic [23]. Nevertheless, there are few studies in the current literature using the OA_m_ in adults with DS to treat snoring and obstructive sleep apnea based on caregiver observations. Yoshida [24] showed a great decrease of respiratory events in patients with cerebral palsy and DS treated with the OA_m_, and stated that this a promising option for this population who usually refuse the use of continuous positive airway pressure (CPAP).

In addition to hypotonia and respiratory sleep disorders, muscle weakness, and slowness in information processing and motor response and in sensory alterations, usually linked to DS, may impair the capacity of these individuals to keep an adequate muscle balance. Consequently, the movements became difficult, slow, and disorganized, also altering swallowing and raising the risk of aspiration pneumonia, thus resulting in feelings of stress, anxiety, and frustration from the usual demands of daily tasks. Therefore, the effects of stress and/or poor sleep quality may trigger an increase in cardiovascular tone, alter heart rate variability, and generate an imbalance of microorganisms in the oral cavity [25, 26]. In addition, analysis of the salivary parameters is a valuable tool to evaluate susceptibility to stress and the lack of balance of microorganisms present in the saliva [27–30].

### Aims and hypotheses

This study aims to assess the physiologic sleep variables, masticatory functions of the bilateral masseter and anterior temporalis muscles, and salivary parameters by means of PSG, sEMG, and specific salivary tests (levels of cortisol, presence of *Pseudomonas aeruginosa*, salivary flow, and pH) respectively, prior to and after neuromuscular electrical stimulation (NMES), the mastication apparatus (MA), and the mandibular advancement oral appliance (OA_m_) in patients with DS. It is hypothesized that oxyhemoglobin desaturation, caused by sleep apnea events, can lead to harmful functions of the neuromuscular system in patients with DS, reducing muscle tonus, which in turn may facilitate obstruction of the upper airways. Moreover, sleep breathing disorders may also lead to sleep architecture fragmentation and increase the arousal index, causing fatigue, nonrestorative sleep, excessive daytime sleepiness, learning and memory difficulties, which may result in low performance in daily tasks, and alterations in the physiological patterns of the various systems of the organism, creating a scenario for the emergence of several comorbidities, including cardiovascular disease that is very common in these individuals. Looking at these problems, it is expected that the OA_m_ will promote a dimensional increase in the oral pharynx during sleep, favoring the airflow and allowing the elimination and/or reduction of snoring and of obstructive sleep apnea. The electrical activity of the masticatory muscles may also be modulated, contributing to reduce the nocturnal obstruction. Furthermore, it is expected that NMES and the MA will contribute to the balance of masticatory muscular functions by means of recruiting muscle fibers. This muscular recruitment will reduce hypotonia and adjust the functions to the physiologic patterns of electrical muscle activity, both in rest and in isometric positions [31, 32]. The samples will be divided according to the randomization rules into three groups: neuromuscular electrical stimulation (NMES), which will be applied to the individuals in Group 1 (DS-NMES); mastication apparatus (MA), which will be applied to individuals in Group 2 (DS-MA); and mandibular advancement oral appliance (OA_m_), which will be applied in individuals in Group 3 (DS-OA_m_). All clinical interventions will be carried out during a period of 2 months.

## Methods/design

**Fig. 1:**
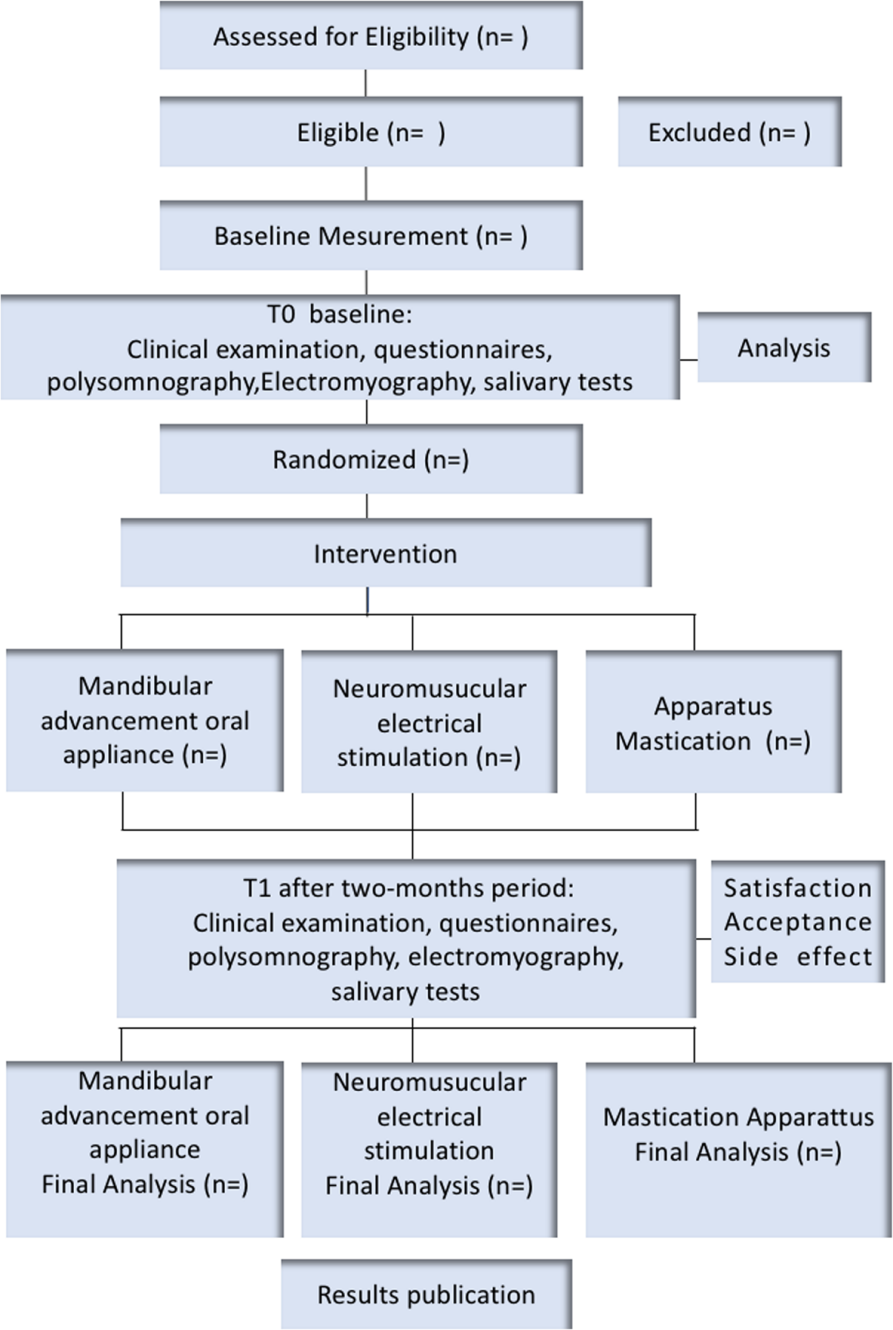
Flowchart of study protocol

**Fig. 2:**
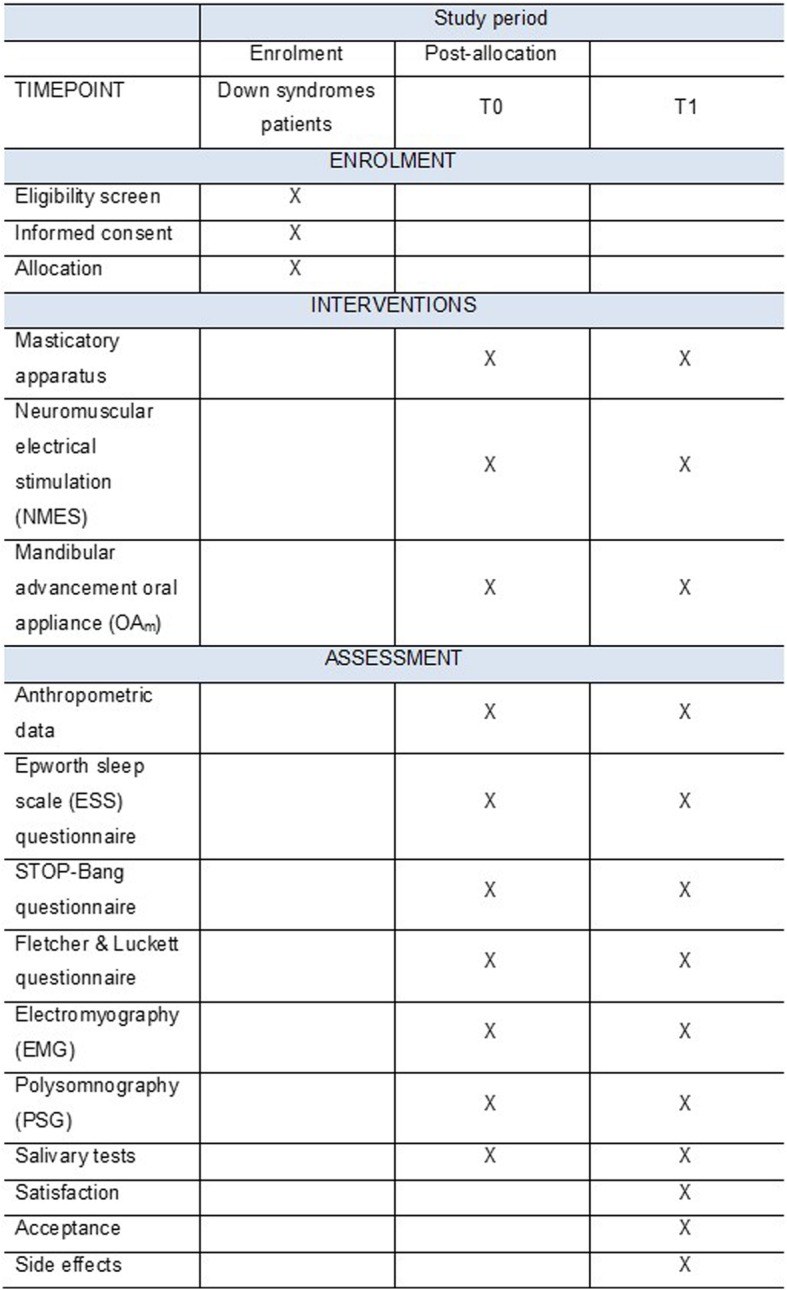
SPIRIT figure: schedule for enrolment, interventions, and assessment. SPIRIT Standard Protocol Items: Recommendations for Interventional Trials

This is a randomized, controlled, three-arm clinical trial conducted according to the ethical standards established in the 1961 Declaration of Helsinki (as revised in Hong Kong in 1989 and in Edinburgh, UK in 2000). This study was registered in the World Health Organization Universal Trial (UTN; number U1111-1201-3155) and Registro Brasileiro de Ensaios Clínicos (ReBEC; number RBR-3qp5np). It was also approved by the Brazilian Human Research Ethics Committee of the Institute of Science and Technology (CEPh 2.127.141; process number CAAE 64173616.4.0000.0077). All caregivers will be informed about the proposal therapies and will provide written informed consent. The whole study design (Fig. 1) and the SPIRIT checklist(aditional file 1) were followed. In addition, a SPIRIT figure is provided to illustrate the study schedule for the enrolment, interventions, and assessment (Fig. 2).

### Subjects

Adult patients with Down syndrome (DS), aged between 18 and 40 years, will be recruited from institutions for people with disabilities. The survey will be conducted at the Center for Bioscience Applied to Patients with Special Health Care Needs (CEBAPE), Institute of Science and Technology—UNESP/SJC, Brazil. The study will include adult patients with DS, from both genders, presenting subjective sleep disorder symptoms, snoring, and/or apnea/hypoapnea index (AHI) > 5.0/h, satisfactory dental health, and preserved cognitive function which allows them to understand and to respond to verbal commands necessary to perform this research project, and who agree to participate by free will including an Awareness Term signed by the patient or by the people responsible for the patient. The researchers will obtain informed consent from assistants, patients, and/or caregivers prior to the study. Cognitive analysis will be carried out under simple commands, such as “open your mouth”, “close your mouth”, “bite”, and “relax”. If patients are able to answer these simple commands, then they will be considered as part of the study. The exclusion criteria include body mass index > 30, use of psychiatric medication, inability to reach the university when needed, signs of psychiatric disease, and having been exposed to physiotherapy and/or orthodontic treatment at least 6 months prior to the beginning of this study.

### Randomization

After evaluation of the eligibility criteria, the subjects will be randomly distributed into the three intervention groups. Randomization numbers will be generated using envelopes, which will contain a card stipulating to which group the subject will be allocated. The envelopes will be sealed and opaque in texture, in order to ensure confidentiality. Participants will be allocated, enrolled, and assigned to the study by two assistants who will not participate in the intervention stage. Participants will be contacted by telephone.

### Sample size

Power analysis or Power test is the process used for determining the sample size for this study, using SPSS (version 22.0.0.0). This is based on a work entitled “Effects of Neuromuscular Electrical Stimulation on the Masticatory Muscles and Physiologic Sleep Variables in Adults with Cerebral Palsy: A Novel Therapeutic Approach” [15]. The intervention was considered to result in a minimum effect similar to the EMG activity of the masseter and temporalis muscles in disabled adults. Thus, the sample size obtained revealed that 90% reliability to identify a relevant outcome will be present at the bidirectional α level of 0.05 with 10 subjects deemed necessary in each group, to which five individuals will be added to compensate for possible dropouts. Due to the risk of withdrawal, 45 patients will be recruited.

### Study interventions

#### Clinical evaluation

Anamnesis will be performed in order to investigate the overall health, treatments, results, and prescription of drugs, medical and family history, parafunctional habits, and psychological aspects. Specific questionnaires will be applied to screen the risk of sleep breathing disorders, sleep quality, and excessive daytime somnolence, including the STOP-BANG, the Epworth Sleep Scale, and the Fletcher & Luckett questionnaires. These questionnaires also comprise the assessment of important sleep features and alterations such as time in bed, sleep time, snoring, gasping and drooling during sleep, the occurrence of nightmares, movement during sleep, and the appraisal of mood at awakening, among other questions. An oral examination will be carried out to address anthropometric data, dental and oropharyngeal status, including the evaluation of dental occlusion, tooth wear severity, number of lost teeth, the degree of tongue scalloping, and modified Mallampati and tonsil scores [33–35].

#### Electromyographical parameters

**Fig. 3:**
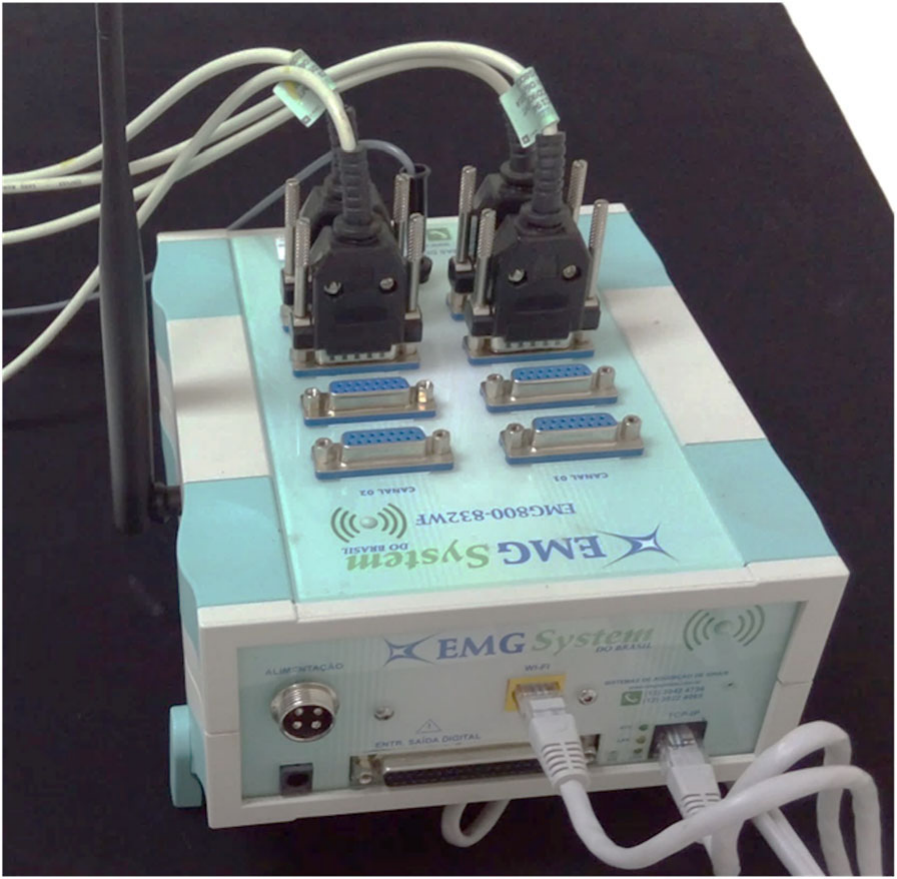
Eight-channel electromyograph (EMG System do Brasil) equipment

The eight-channel module (EMG System do Brasil) (Fig. 3) will be used to capture biological signals. This equipment consists of a conditioner with a bandpass filter with cutoff frequencies at 20–1000 Hz, an amplifier gain of 1000×, and a common mode rejection ratio > 120 dB. All data will be processed using specific software for acquisition and analysis (EMG System do Brasil), with a 16-bit analog to digital converter and a sampling frequency of anti-aliasing of 2 kHz for each channel. Active bipolar electrodes with a preamplification gain of 20× are included.

**Fig. 4:**
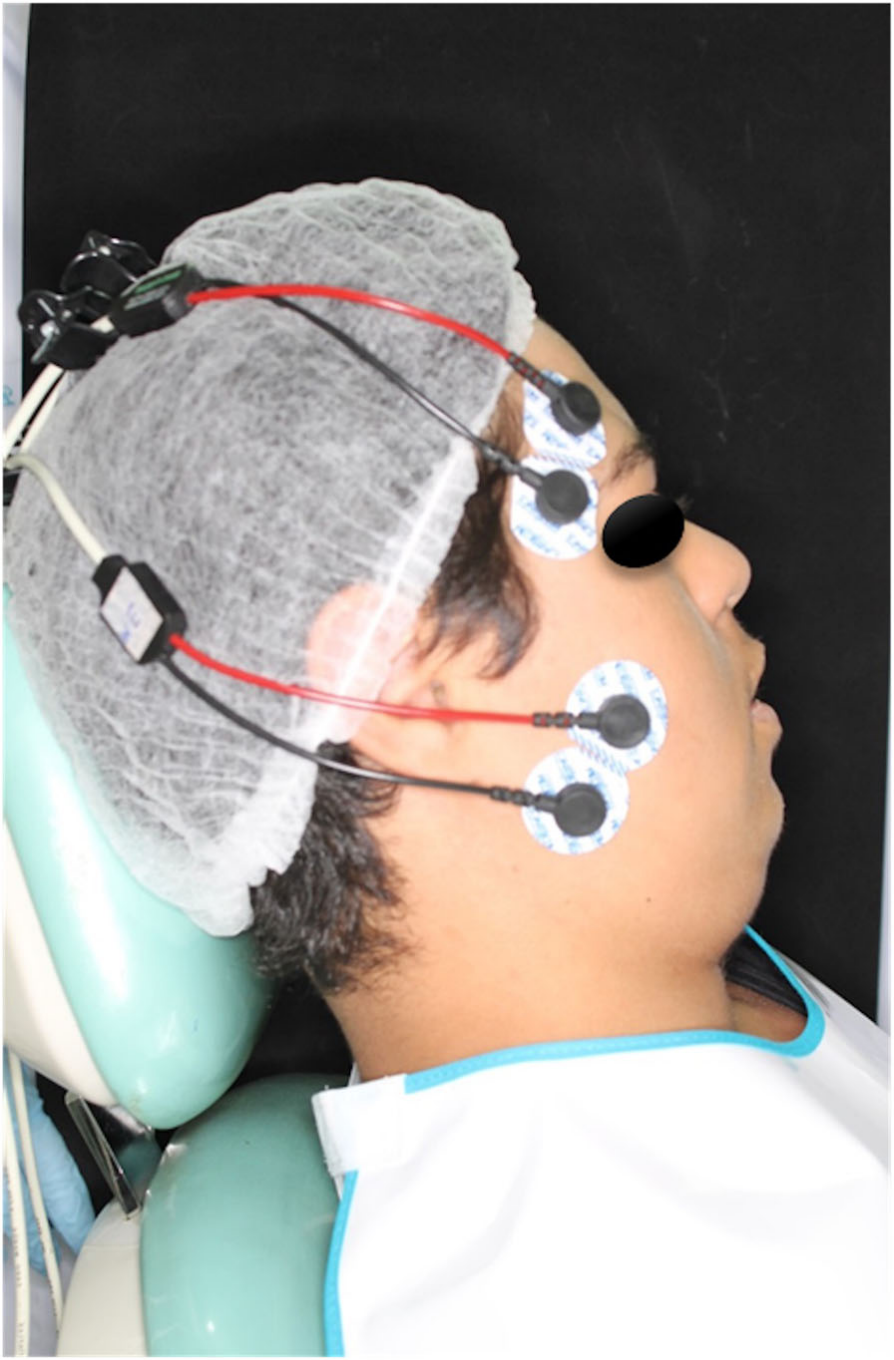
Electrode placement for surface electromyography examination

Surface electromyography (sEMG) of right and left masseter (RM and LM) and right and left anterior temporalis (RT and LT) muscles will be acquired prior to application of the mandibular advancement oral appliance (OA_m_), the mastication apparatus (MA), and neuromuscular electrical stimulation (NMES), as well as 2 months after all therapies. During the sessions, the patient will be instructed to remain seated on a chair, feet apart, shoulders relaxed, and hands resting on their thighs, with their head turned to the Frankfourt parallel to the ground and no visual feedback of the signals displayed on the computer, in a well-illuminated, silent recording room in a comfortable position, with both eyes open. A short training period will be conducted prior to beginning the tests to familiarize the patient with the tasks. The skin of the patient will then be cleaned with a cotton ball soaked in 70% alcohol to reduce impedance. Next, pregelled, self-adhesive, bipolar, disposable 10-mm-diameter Ag/AgCl surface electrodes (MediTrace®) will be fastened over the right masseter (RM), left masseter (LM), right temporalis (RT), and left temporalis (LT) muscles, with an interelectrode distance of 10 mm, on the muscle motor point, after the patient has performed moderate intercuspation. Surface electrodes will be bilaterally fastened according to anatomical references and guided by the direction of muscle fibers at two points: the anterior temporal muscle, 2–3 cm superior–posterior distance to the lateral corner of the eyes in the region of greatest evidence of muscle mass, parallel to the muscle fibers, but with its sensing surface perpendicularly oriented; and the superficial portion of the masseter, 1–2 cm above the gonial angle of the mandible, in the region of greatest evidence of muscle mass, with muscle fibers parallel to the surface (Fig. 4). Also, a grounding electrode (Ag/AgCl 10-mm-diameter surface electrode; MediTrace®) will be fastened to the left wrist of the patients.

#### Analysis of surface electromyography data

In order to obtain a frequency domain analysis, surface EMG signal processing will be carried out using specific routines conducted with the Matlab program, version 7.1 (The MathWorks Inc., Natick, MA, USA). In addition, the time domain analysis will be executed using the root mean square (RMS) of the EMG signal (expressed in μV) and will be normalized by the highest values of RMS obtained during the three MHI and MVC readings (μVmV/μV/100: % MVC). The data captured at rest (total of 15 s) will be used for both analyses during MHI and MVC (total of 3 s; the first and fifth seconds having been discarded).

#### Protocols for surface electromyography, mandibular force, and mouth opening

**Fig. 5:**
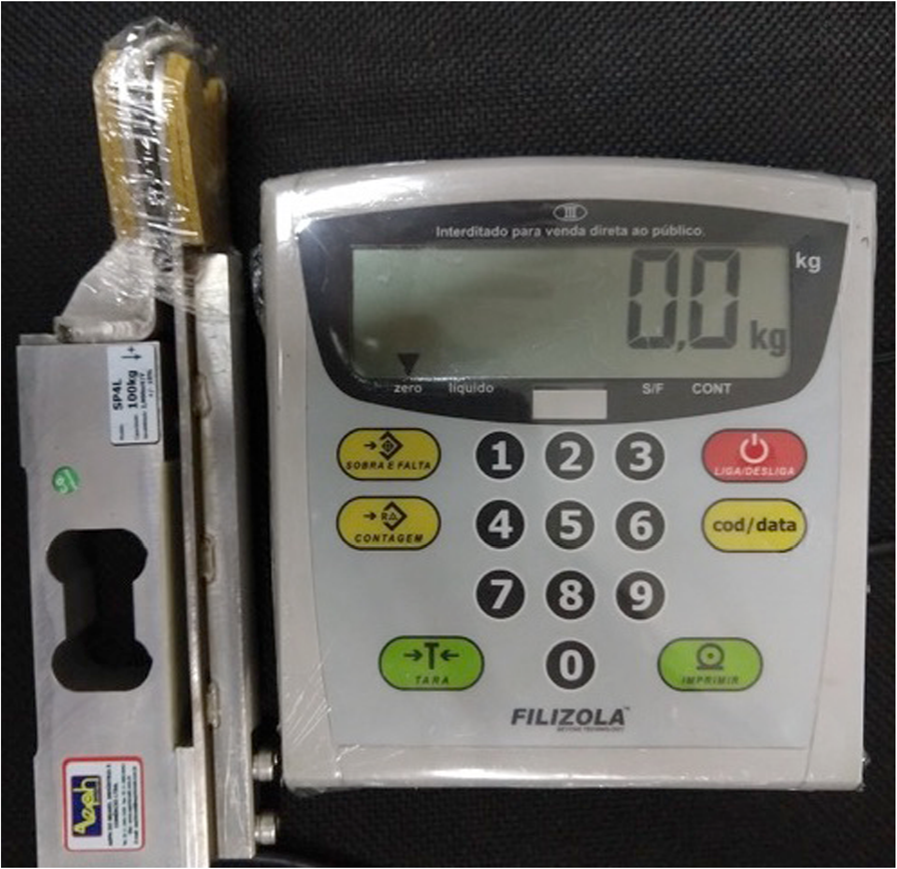
Mandibular force transducer (EMG System do Brazil)

**Fig. 6:**
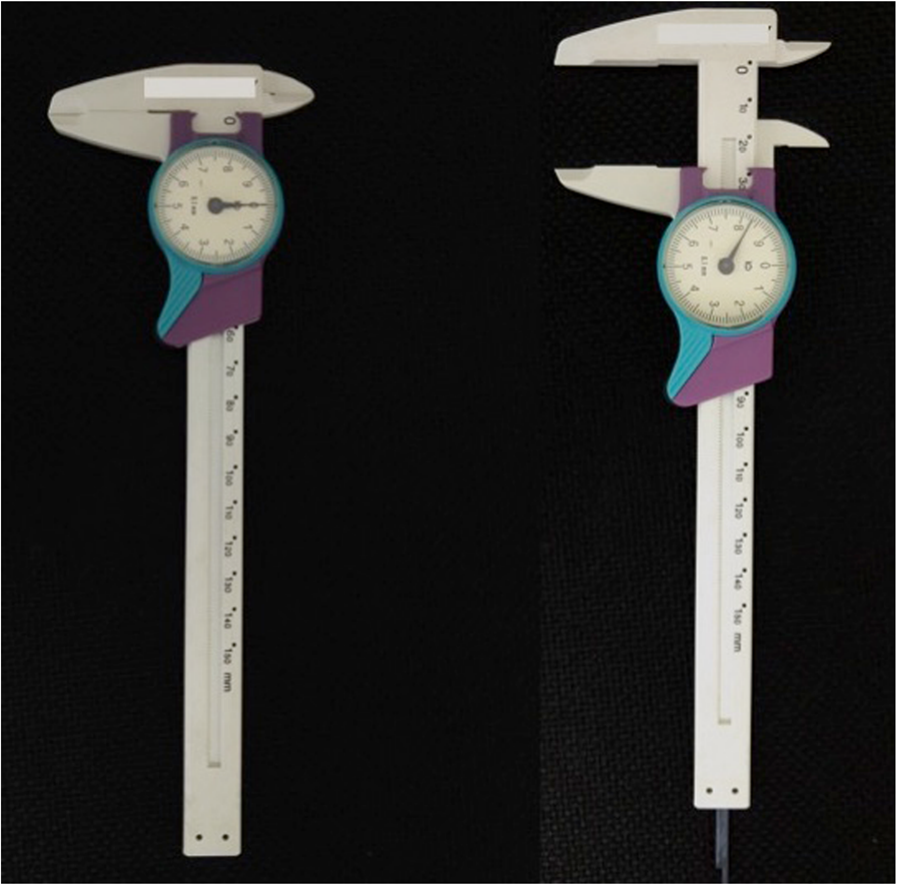
Caliper to measure mouth opening

The selected protocol for sEMG is based on previous work [13, 15, 36]. The sEMG records will be acquired under the following conditions: rest (three sequential readings of 10 s each); maximum voluntary contraction (MVC) with cotton balls placed between the upper and lower molars on each side (three readings of 5 s each, with a 2-min interval between the readings); and voluntary contraction with maximum habitual intercuspation (MHI) with no material placed between the teeth (three readings of 5 s each, with a 2-min interval between the readings). After a 3-min interval, a mandibular force transducer (EMG System do Brazil) (Fig. 5) will be used to record the maximum bite force. This consists of a mechanical device with sensors that record material deformations during the bite. These deformations are converted into kilograms-force or Newtons by means of EMG Lab V1.1 software (EMG System do Brazil). The maximal opening amplitude will be measured with a specific caliper (Fig. 6).

#### Phases of the surface electromyography examination

The sEMG, referring to the bilateral masseter (surface portion) and to the temporal (anterior portion) muscles in rest, MVC, MHI, and bite force, shall be carried out in two distinct phases: Phase 1, initial data acquisition, prior to proposed therapies (Groups 1–3); and phase 2, treated groups (Groups 1–3) after 2 months of neuromuscular electrical stimulation, mastication apparatus with mastication instrument Hiperboloide®, and mandibular advancement oral appliance therapies. The obtained data will be compared among each group and among each person with themselves, to verify the effectiveness of the proposed therapies on the improvement of the masticatory muscle electrical activity, on the physiological sleep variables, and on salivary parameters in patients with DS.

#### Protocol for polysomnography

All patients will undergo the PSG type II home sleep test (Embla Embletta MPR + PG ST + Proxy; Natus, CA, USA) in their own house. The PSG will be performed prior to and after 2 months of all proposed therapies. During the night, a sleep technician will visit each patient’s home for examination preparation. All recording sensors will be placed according to recommended PSG techniques, using a gel-covered transducer, tapes, or elastic straps. The following biological signals will be recorded simultaneously and continuously: six channels for the electroencephalogram (EEG) (F4–M1, C4–M1, O2–M1, F3–M2, C3–M2, O1–M2), two channels for the electrooculogram (EOG) (E1–M2, E2–M2), four channels for the surface electromyogram (muscles of the submentonian region, anterior tibialis muscle), one channel for the electrocardiogram (derivation V1 modified), two channels for airflow (oronasal thermistor, nasal pressure transducer), two channels for respiratory effort (chest and abdomen, via x-trace belts), one channel for snoring, and one channel for body position, via EMBLA sensors. Arterial oxygen saturation (SaO_2_) and heart rate will be recorded via an EMBLA oximeter. All sleep staging and EEG arousals, respiratory events, and leg movements will be visually scored according to the *American Academy of Sleep Medicine (AASM) Manual for the Scoring of Sleep and Associated Events*, version 2.2, which is the definitive reference for standardized sleep monitoring and scoring [37]. The patients will be instructed to remain as relaxed as possible and to sleep naturally, as if at home. All signals will be recorded continuously. In the morning, an experienced polysomnography technician will remove the PSG equipment from the patient. A sleep physician will read and interpret all PSG examinations.

#### Protocol for salivary parameters

##### Saliva sample collection

Five steps will be defined in order to analyze the physical (determinations of the salivary flow and viscosity), chemical (buffer capacity and cortisol levels), and microbiological (levels of *P. aeruginosa*) properties of the saliva. In the first step, the patient must ingest no drink or foods for 2 h before saliva collection. The second step is rinsing the mouth thoroughly with distilled or deionized water (20 ml) for 20 s and collecting saliva samples 30–40 min after rinsing. The third step is to analyze the physical properties; a gustatory sialagogue will be applied as drops on the back of the tongue for 5 min, at an interval of 4 drops/min, to stimulate the saliva in patients with muscular hypotonicity and cognitive deficit. In order to analyze the chemical properties, the digital mechanical stimulation or milking of the major salivary glands will be carried out for 5–10 min. In the fourth step, simultaneously, the salivary flow will be aspirated by means of a low-power suction device (Aspiramax MA 520; NS, São Paulo, Brazil) and placed in a graduated sterile test tube which will be refrigerated at a temperature of 4 °C. In the fifth step, the saliva samples will be stored in a container at a temperature of 4 °C and later transported to the genome laboratory. In order to ensure identification of the *P. aeruginosa* species, the saliva sample must be analyzed within a maximum period of 3 h.

#### Analysis of the physical salivary properties of the saliva

##### Method for measurement of the saliva flow

After comfortable and postural adaptation of the patients on the chair, the saliva will be stimulated through a gustatory sialagogue, and then continuously collected for 5 min by means of a Falcon-type sterile tube which will be coupled to an aspirating pump. The saliva flow (SF) will be calculated by the formula: SF = total salivary volume / salivary collection time. The reference values are described in the study by Gomes et al. [38].

##### Method for measurement of the salivary viscosity

Saliva viscosity (texture) will be considered as this physical property strongly helps in regular swallowing. In light of this, the viscosity will be evaluated by means of an Ostwald viscometer, model Cannon-Fenske Routine, size 350, internal capillary with a caliper of 1.52 (Hermex Indústria e Comércio de Vidros para Laboratório, São Paulo, Brazil). Reference values (centipoise) are < 1.5 cp for nonviscous saliva and ≥ 1.5 cp for viscous saliva. In order to standardize the testing temperature, the viscometer will be vertically positioned by the “L” tube in a thermal bath at 37 °C for 15 min. A sample of 5 ml of saliva samples will then be inserted into the “N” tube of the Ostwald viscometer, using a hypodermic syringe. Concomitantly, aspiration will be carried out by inserting the pear into the “L” tube opening to force the saliva toward the “E” mark of the “C” bulb. Thereafter, the vacuum will be released and the saliva outflow time, between the “E” and “F” marks, will be clocked. This methodology is described in the studies by Almeida et al. [39] and Pedersen et al. [40].

#### Analysis of the chemical salivary properties of the saliva

##### Method for measurement of the buffer capacity

In this test, a 1 ml sample of saliva will be transferred to a sterile Falcon-type tube. Next, 3 ml of hydrochloric acid (0.005 N) will be added to this sample. The tube will be lightly shaken and allowed to rest for 10 min. Immediately thereafter, the pH will be measured with the aid of a pHmeter (DM-22 model; Digicrom Analítica Ltda., São Paulo, Brazil). Reference values are described in the study by Gomes et al. [38].

##### Method for measurement of cortisol levels

Cortisol will be the physiological biomarker used to assess the susceptibility to psychological and/or physical stress in the saliva samples. The changes in cortisol levels usually follow the circadian rhythm, which includes three phases. The first phase is the response to the cortisol awakening (RCA). This phase is defined as a high increase in cortisol levels within 30 min after awakening, followed by a high decline within the following 3 h. The second phase shows a gradual decline in cortisol during the rest of the day, reaching the lowest concentration during the first half of sleep. Finally, the third phase corresponds to a gradual increase in cortisol levels until awakening [41, 42].

Therefore, considering that cortisol secretion is regulated by the circadian cycle and that the salivary cortisol concentration is directly related to the degree of activation of the hypothalamic–pituitary–adrenal (HPA) axis [43], the authors hypothesize the existence of variability in cortisol levels in the saliva of patients with DS. Therefore, the cortisol levels (susceptibility to stress) will be assessed in two different periods, in the morning and at night. Reference values for salivary cortisol levels in the morning and at night are respectively < 0.78 μg/dl and < 0.20 μg/dl, in accordance with the recommendations of Castro and Moreira [43].

The level of salivary cortisol will be dosed by means of an enzyme immunoassay kit (product #1-1102; Salimetrics, State College, PA, USA) used at room temperature (25 °C). The sample volumes will be 25 μl with an incubation time of 60 min. The controls and the standards will be dosed in the same plate. The procedure follows the basic principle of the enzyme immunoassay, wherein competition occurs between an unlabeled antigen and an antigen labeled with enzyme by a determined number of binding sites on the antibody. The test set will be carried out on the microtiter plate which is coated by monoclonal antibodies to cortisol. The standard and unknown cortisol compete with the wild-rabbit peroxidase-linked cortisol. The wild-type rabbit isolated enzyme acts as an antigen by the antibody binding sites. After incubation, deactivated components will be removed by washing. Peroxidase-linked cortisol will be measured by the peroxidase enzyme reaction on the tetramethylbenzidine (TMB) substrate. This reaction will produce a blue color. A yellow color will be formed after the reaction is finished, with the addition of sulfuric acid. Optical density analysis will be performed by the absorbance reading of the solution at 450 nm (correction filter from 490 to 630 nm) (EON, Biotek Instruments, Inc., Winooski, VT, USA). The amount of peroxidase cortisol, which will be detected by means of color intensity, is inversely proportional to the amount of present cortisol.

#### Analysis of the microbiological properties of the saliva

##### Method for measurement of *Pseudomonas aeruginosa* species

The methodology for the isolation and identification of *P. aeruginosa* will be performed to evaluate the risk of occurrence of aspiration pneumonia. No cleaning of the tongue must be accomplished by the patients with DS for at least 24 h. The mouthwash samples, which will be collected in physiological solution, will be centrifuged, resuspended, and seeded, in duplicate, in Petri dishes containing MacConkey agar (Difco, Detroit, MI, USA) for the isolation of enterobacteria/pseudomonas. The plates will be incubated at 37 °C for 48 h. Thereafter, the number of colony-forming units per milliliter (CFU/ml) will be calculated. Colonies with different morphologies will be stained by the Gram method. Five colonies of Gram-negative bacteria will be isolated in agarose gel and incubated at 37 °C for 24 h. Then, the tubes will be stored at 4 °C. The enterobacterial/pseudomonas strains will be identified by the API20 E system (Bio-Merieux, France), aiming at the detection of *P. aeruginosa* species. This methodology is described in the study by Pereira et al. [44].

#### Protocol for neuromuscular electrical stimulation

**Fig. 7:**
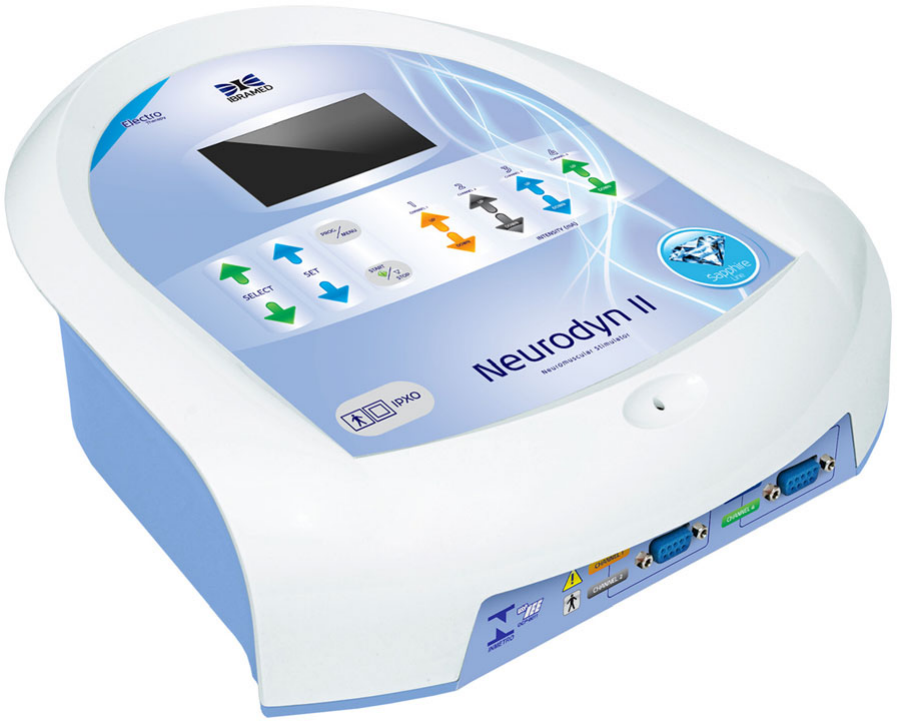
Four-channel neuromuscular electrical stimulation (Neurodyn II Ibramed) equipment

Neuromuscular electrical stimulation (NMES) will be used to improve and/or balance the muscular tonus and to modulate masticatory and oropharyngeal muscle functions. This method has proven to be a safe and effective therapy [15]. The Neurodyn Imbramed II equipment with a four-channel device will be used (Fig. 7). NMES is a noninvasive, safe, and effective treatment, which uses electrical impulses to stimulate and/or modulate a group of injured and/or altered muscle functions. It can also favor blood circulation, improve muscle spasms, reduce edema caused by inflammatory processes, and increase muscle strength, resulting in gain of the muscular tonus [20, 21]. The equipment will deliver a low-current intensity in order to induce action potentials in motor nerves, resulting in the activation of motor units [45, 46]. This therapy produces electrical stimulation on a group of muscles, through surface electrodes correctly placed on the muscles. NMES will be performed according to Guidelines 601 and 601-2-10 of the International Electrotechnical Commission, which establishes the rules for the respective usage of electromedical devices and electrical stimulators. The surface electrodes will be placed on the muscle motor point (bilateral masseter and anterior temporalis). The following parameters will be used: pulse frequency of 50 Hz, pulse width of 250 μs, on/off ratio of 5 s of stimulation and 10 s of rest for 20 min, and low-current intensity (range from 12 to 19 mA) per session, depending on patient sensitivity, and according to the protocol developed by Giannasi et al. [14, 15]. The patients will undertake three 20-min sessions per week of NMES on the bilateral masseter and the anterior temporalis muscles during a 2-month period. The described parameters may evoke muscle response with minimal discomfort to the patients, although most patients usually do not mention feeling any discomfort or pain. A visible muscle contraction will be noted when NMES is applied, meaning that the electrical stimulus produced the necessary contraction.

#### Protocol for mastication apparatus

**Fig. 8:**
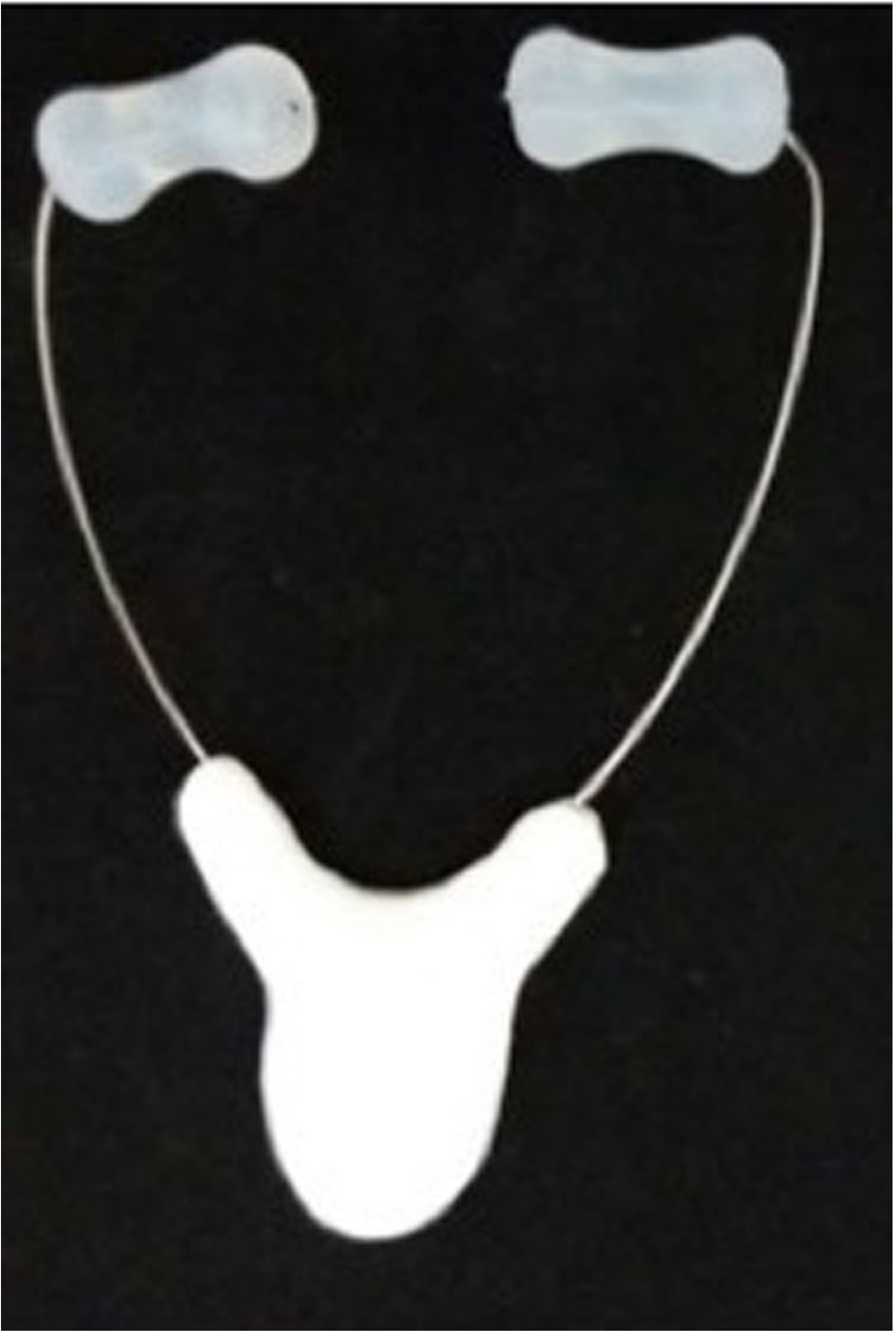
Hyperboloid® fixed to the support rod

The mastication apparatus (MA) will be used to strengthen, stimulate, and modulate the masticatory muscles, showing a safe, good cost–benefit, and effective myofunctional approach [13, 47]. This MA is composed of two mastication instruments, Hiperboloide® (M.C. CHEIDA—ME, São Paulo, Brazil; http://www.hiperboloide.com), which are fixed in a support rod (SR). This SR is formed from a body of compact or fenestrated thermoplastic material and a stainless-steel wire with two free distal tips to couple the hyperboloids (active tips) (Fig. 8), which was developed by the transdisciplinary team of the CEBAPE-UNESP (the patent process is in progress, under protocol number 18CI038). Mastication apparatus therapy will be performed for 5 min, six times per day, with a minimum interval of 2 h between sessions, and for a period of 2 months. The individuals will simultaneously bite into the two active tips of the MA for 3 s and will release it for 1 s for a total duration of 5 min. This protocol was developed by Giannasi et al. [13]. These patients will be instructed to bite the MA in a slow rhythm. The therapy must be interrupted if the patient complains about feeling pain. After the mechanical exercises, the patients and/or caregivers will be instructed to clean the support rod with running water to remove residual remnants, dry it, and then store it in a safe, fresh, and clean location.

#### Protocol for the mandibular advancement oral appliance

The mandibular advancement oral appliance (OA_m_) is one of the most commonly used therapies to treat OSA safely and effectively. It is comfortable, silent, portable, and well tolerated. The appliance will protrude the mandible and base tongue, leading to an increase in upper airway dimensions. All of this alteration allowed by mandibular protrusion will promote the reduction/elimination of snoring and other respiratory events, as well as associated symptoms [48]. OA_m_ fabrication will be based on the definition of an effective device to treat OSA published by the American Academy of Dental Sleep Medicine. The OA_m_ features areas are follow - custom fabricated, allow titration (gradual mandibular advancement) and lateral movement, occlusal coverage, easy and suitable placement and removal by the patient or caregiver, and retentive relationship with the teeth [37]. The patients will use the oral appliance only during sleep, for a 2-month period. The OA_m_ titration will be done with increments of 0.25–1.0 mm, weekly or biweekly. The appliance will be constructed with acrylic resin in two parts (one for the upper arch and one for the lower arch), with complete coverage of the occlusal sides of the teeth, joined together by a hyrax 13-mm expansion screw on each side. The OA_m_ will be fitted and instructions on how to use the device will be given. The titration will be performed based on the needs of each individual, with mandibular advancement of 0.25-mm increments per turn [49]. The amount of advancement will be done based on reports by the patient and their caregivers regarding reduction in snoring, sleep apnea events, and initial symptoms and/or based on patients’ physiological limitations according to the advancement protocol developed by Giannasi et al. [49]. Not always is a great amount of protrusion necessary [50].

**Fig. 9:**
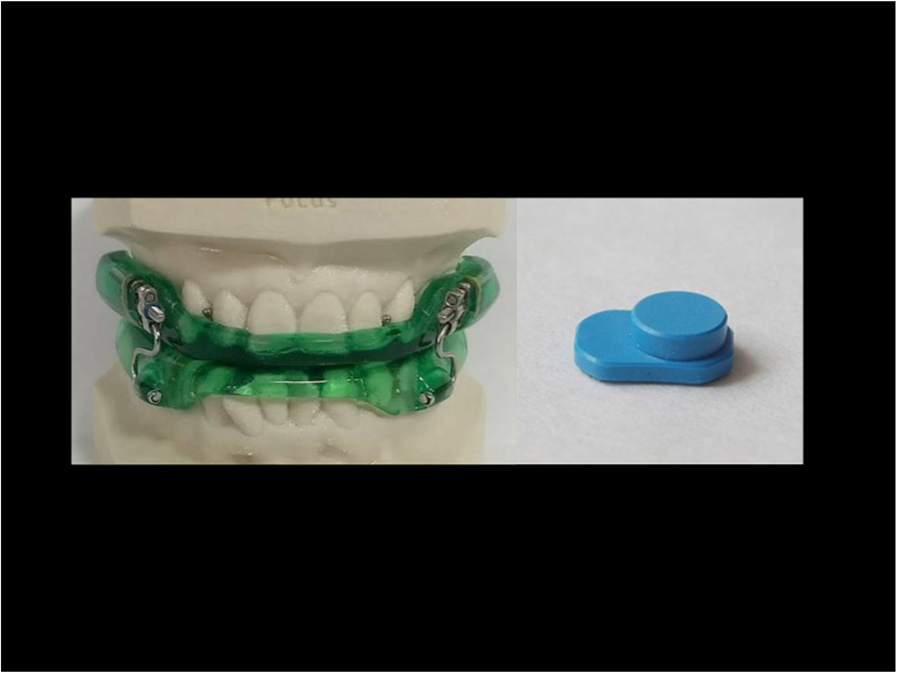
Mandibular advancement oral appliance and thermosensitive microchip TheraMon®

Patients will use the OA_m_ with an embedded temperature-sensitive microsensor with on-chip readout integrated electronics TheraMon® (Fig. 9). The data recorded in the chip will be read through a dedicated reading station. This microsensor will allow the measurement of objective OA_m_ compliance, based on the assumption that the oral appliance was worn when a temperature > 35 °C was recorded. The objective measurement is expressed as the objective mean rate of OA_m_ usage in number of hours used per day, and as the percentage of days of OA_m_ usage per week [39, 40]. The patient will be considered compliant when OA_m_ usage > 4 h per night. This information allows for calculation of the Mean Disease Alleviation (MDA), as a measure of therapeutic effectiveness [51–53]. The MAD, expressed as a percentage, is calculated as: MAD = [(adjusted objective × therapeutic efficacy) / 100].

The MAD concept in the dental sleep field has been used to evaluate the therapeutics effectiveness of OA_m_ therapy, which is compounded by efficacy (immediate result) plus adherence to treatment over time [39–41]. The measurement of objective compliance is important because it is necessary to know if any therapy continues to have positive results over time. With this information it is possible to determine the MDA and so the therapeutic effectiveness. The meaning of the effectiveness of any therapy is compounded by efficacy plus adherence to such treatment over time [51–53].

### Quality control

In order to ensure data quality, the dentists responsible for performing the EMG examination, as well as the sleep technicians responsible for the polysomnography examination, will receive appropriate training. External monitoring will be carried out periodically to verify the performance of EMG and polysomnography.

Guidance on the correct use and performance of each therapy will be given at each appointment, and all participants/caregivers will have access to an exclusive telephone number to solve any doubts they may have during the study, in order to avoid any misunderstanding that may compromise the results. This telephone contact will also be an instrument to reinforce the given guidelines and to encourage adherence to the therapies.

It will be explained to the participants/caregivers that they will be excluded whenever physiotherapy or speech therapy is to be undertaken during the study.

In addition, all participants/caregivers will be aware that they are free to withdraw their participation from this study at any time if they so wish, with no penalties.

If the participants face any difficulties to perform the proposed therapy, then they will be allocated to another therapy that they are able to perform.

The data obtained prior to and 2 months after all proposed therapies will be analyzed by “blind” evaluators. In addition, satisfaction, acceptance, and side effects will be assessed after a 2-month period, for each therapy applied.

### Statistical analysis

Whenever applicable, the data will be presented as average ± standard deviation. The paired *t* test will be used in order to compare continuous variables with normal distribution, prior to and after polysomnography, as well as specific therapies. If the data show a non-normal distribution, then the Wilcoxon test will be used. The groups will be compared to one another using ANOVA or Mann–Whitney U test, according to the distribution. *P* ≤ 0.05 will be considered statistically significant. Missing data will be treated as worst-case scenario imputation. All analyses will be performed using SPSS 16.0.

All data will be stored on a notebook exclusively for this purpose, as well as on a hard disk for increased safety of data storage. Data will be allocated to a file with a number that corresponds to each participant and will be managed by the assistants, not available to the professionals who will carry out the interventions.

## Discussion

This clinical trial will assess the physiologic sleep variables, masticatory function of the masseter and anterior temporalis bilateral muscles, and salivary parameters by means of PSG, sEMG, and specific salivary tests (cortisol levels, presence of *P. aeruginosa,* salivary flow, and pH) respectively, prior to and after mandibular advancement oral appliance (OA_m_), neuromuscular electrical stimulation (NMES), and mastication apparatus (MA) therapies in patients with Down syndrome. As the primary outcome, the authors believe that OA_m_, neuromuscular electrical stimulation, and mastication apparatus therapies may improve masticatory and oral pharynx muscle modulation, adjusting them to a level close to normality, which may lead to a balanced muscle activity contributing to reduce snoring/sleep apnea. Good sleep quality is likely to reduce corporal stress, as shown by the decrease in cortisol levels in the saliva, as a secondary outcome. In addition, the authors expect an improvement in other salivary parameters, as the absence of snoring and/or sleep apnea, which result from the act of breathing through their mouth, will contribute to the balance of the microorganisms in the oral cavity. Moreover, polysomnography will be used to evaluate the other sleep variables and the authors believe that sleep quality will improve after all therapies have been carried out. Another expected outcome is to measure the objective compliance and adherence to the OA_m_ with an embedded microsensor thermometer.

It is important to highlight that none of these therapies will cause any injuries to the participants, so the hypothesis of harm or of adverse side effects does not apply to the present study.

Neuromuscular electrical stimulation (NMES) may produce a feeling of discomfort due to the electrical stimulus applied on masticatory muscles; however, it does not offer foreseeable risks. The mastication apparatus (MA) does not produce discomfort or foreseeable risks. The mandibular advancement oral appliance (OA_m_) embedded with a thermosensitive microchip may initially produce discreet dental and muscular discomfort, with possible temporary hypersalivation and risks of occlusal changes. The polysomnography, surface electromyography, and specific salivary tests do not produce discomfort or foreseeable risks.

These proposed therapies may contribute to an improvement in speech, swallowing, and mastication, as well as an improvement in physiologic sleep variables such as oxyhemoglobin desaturation, REM sleep, and deep sleep, and contribute to the reduction of snoring and sleep apnea. We hypothesize that this fact occurred due to cascade effects from stimulation of masticatory muscles (masseter and temporalis muscles), initiating an activation of the upper airway muscular chain, including the oropharyngeal, velopharyngeal, hypopharyngeal, and tongue base muscles. Thus, all these therapies may promote improvements in the stomatognatic system performance. In addition, salivary parameters may also show significant improvement.

## Additional file

Additional file 1: SPIRIT 2013 checklist: recommended items to address in a clinical trial protocol and related documents (DOC 120 kb)

## Abbreviations

DS: Down syndrome
MA: Mastication apparatus
NMES: Neuromuscular electrical stimulation
OA_m_: Mandibular advancement oral appliance
OSA: Obstructive sleep apnea
PSG: Polysomnography
sEMG: Surface electromyography
SR: Support rod
